# Neurovascular Coupling Is Impaired in Hypertensive and Diabetic Subjects Without Symptomatic Cerebrovascular Disease

**DOI:** 10.3389/fnagi.2021.728007

**Published:** 2021-10-06

**Authors:** Ana Monteiro, Pedro Castro, Gilberto Pereira, Carmen Ferreira, Farzaneh Sorond, Andrew Milstead, James P. Higgins, Jorge Polónia, Elsa Azevedo

**Affiliations:** ^1^Department of Clinical Neurosciences and Mental Health, Faculty of Medicine of University of Porto, Porto, Portugal; ^2^Department of Neurology, Unidade Local de Saúde de Matosinhos, Matosinhos, Portugal; ^3^Department of Neurology, Centro Hospitalar Universitário de São João, Porto, Portugal; ^4^Department of Neurology, Division of Stroke and Neurocritical, Northwestern University Feinberg School of Medicine, Chicago, IL, United States; ^5^Department of Radiology, Northwestern University Feinberg School of Medicine, Chicago, IL, United States; ^6^Hypertension Unit, Unidade Local de Saúde de Matosinhos, Matosinhos, Portugal; ^7^Department of Medicine, Faculty of Medicine of University of Porto, Porto, Portugal

**Keywords:** hypertension, diabetes mellitus, neurovascular coupling (NVC), transcranial doppler (TCD), cerebral small vessel disease

## Abstract

The mechanistic link between hypertension, diabetes and cerebral small vessel disease (CSVD) is still poorly understood. We hypothesized that hypertension and diabetes could impair cerebrovascular regulation prior to irreversibly established cerebrovascular disease. In this study, 52 hypertensive patients [54% males; age 64 ± 11 years; 58% with comorbid diabetes mellitus (DM)] without symptomatic cerebrovascular disease underwent transcranial Doppler (TCD) monitoring in the middle (MCA) and posterior (PCA) cerebral arteries, to assess vasoreactivity to carbon dioxide (VRCO_2_) and neurovascular coupling (NVC). 1.5T magnetic resonance imaging was also performed and white matter hyperintensity volume was automatically segmented from FLAIR sequences. TCD data from 17 healthy controls were obtained for comparison (47% males; age 60 ± 16 years). Hypertensive patients showed significant impairment of NVC in the PCA, with reduced increment in cerebral blood flow velocity during visual stimulation (22.4 ± 9.2 vs. 31.6 ± 5.7, *p* < 0.001), as well as disturbed NVC time-varying properties, with slower response (lower rate time: 0.00 ± 0.02 vs. 0.03 ± 6.81, *p* = 0.001), and reduced system oscillation (reduced natural frequency: 0.18 ± 0.08 vs. 0.22 ± 0.06, *p* < 0.001), when compared to controls. VRCO_2_ remained relatively preserved in MCA and PCA. These results were worse in hypertensive diabetic patients, with lower natural frequency (*p* = 0.043) than non-diabetic patients. White matter disease burden did not predict worse NVC. These findings suggest that hypertensive diabetic patients may have a precocious impairment of NVC, already occurring without symptomatic CSVD. Future research is warranted to evaluate whether NVC assessment could be useful as an early, non-invasive, surrogate marker for CSVD.

## Introduction

Cerebral small vessel disease (CSVD) has an enormous impact on public health worldwide (GBD 2017 Causes of Death Collaborators, 2018). It accounts for 25% of ischemic strokes and most hemorrhagic strokes and is the second leading cause for cognitive decline (Sudlow and Warlow, 1997; Iadecola et al., 2019).

Hypertension (HT) is the major vascular risk factor (VRF) for CSVD. Alongside HT, diabetes mellitus (DM) is a recognized VRF implicated in CSVD (Brundel et al., 2012; Liu et al., 2018).

While other etiologies for stroke are fairly well-studied, the pathophysiology and causality of CSVD are still poorly understood. Many of its manifestations are clinically silent until the development of clinical consequences, with stroke, cognitive decline, and gait impairment, limiting disease-specific preventive strategies (Pantoni, 2010). Also, CSVD radiological markers and clinical manifestations seem to be dissociated, for reasons not fully explained (Sorond et al., 2011; Jokumsen-Cabral et al., 2019). Biomarkers for the events predating irreversible damage could be key for better clinical management and pre-symptomatic preventive measures.

There is evidence of neurovascular dysfunction in CSVD and it may precede clinical and imaging manifestations (Wardlaw, 2010; Freeze et al., 2018; Castro et al., 2020). Very few studies have investigated neurovascular coupling (NVC) in HT, mostly using imaging modalities. Transcranial Doppler (TCD) is a non-invasive method that allows the monitoring of microvascular hemodynamic functional integrity (Claassen et al., 2016; Malojcic et al., 2017).

We aimed to study cerebrovascular regulation by TCD in hypertensive and diabetic patients without major CSVD-related impairment as a possible surrogate marker for the future development of symptomatic CSVD, to help guide therapies aimed at the cerebral microcirculation and neurovascular unit.

## Materials and Methods

### Study Subjects

A cross-sectional observational study was conducted in a University Hospital. Hypertensive patients were recruited from the hospital's Hypertension Unit. Exclusion criteria were previous stroke or other significant brain pathology (dementia by clinical criteria, brain tumor, traumatic brain injury, previous cerebral infection or neurodegenerative disease), severe/unstable disease, contraindication for magnetic resonance imaging (MRI), inadequate acoustic temporal bone window, extra- or intracranial artery stenosis >50% and incapability to collaborate or to give informed consent. TCD data from healthy controls of similar age and gender were obtained from previous studies performed with the same protocol (Jokumsen-Cabral et al., 2019).

The local ethics committee approved the study protocol, which followed the tenets of the Declaration of Helsinki. Written informed consent was obtained.

### Clinical Evaluation

Participant's clinical and demographic data were recorded. Vascular comorbidities were summarized into a vascular comorbidity score (VCS), including HT, DM, dyslipidemia, tobacco usage, chronic heart failure, coronary heart disease, arrhythmias, peripheral artery disease, and nephropathy. These conditions were scored as present (1 point) or absent (0 points), for a score ranging from 0 to 9 (Mossello et al., 2015). All participants underwent cervical and transcranial Doppler ultrasound (Philips iu22, The Nederlands) to exclude hemodynamically significant vessel pathology. The patients underwent routine 24-h ambulatory blood pressure monitoring (Spacelabs 90207, Redmond, Washington, USA). The mini-mental state examination (MMSE) and the Montreal cognitive assessment (MoCA) were used to screen for dementia. The patients were evaluated by an ophthalmologist, and all had normal binocular visual acuity, allowing for the TCD dynamic testing.

### Monitoring Protocol

Evaluations were conducted in a dim-lighted, quiet room (≃22°C), in a supine position. Cerebral blood flow velocity (CBFV) was continuously recorded in the M1 segment of the right middle cerebral artery (MCA) and the P2 segment of the left posterior cerebral artery (PCA), with 2-MHz TCD probes secured with a headframe (Doppler BoxX, DWL, Singen, Germany), in order to simultaneously obtain data from both arterial territories (Azevedo et al., 2012). Continuous non-invasive arterial blood pressure (BP) was measured with the Finometer (FMS, Amsterdam, The Netherlands). Heart rate was assessed with a three-lead electrocardiogram. End-tidal carbon dioxide (EtCO_2_) was recorded by capnography (Respsense Nonin, Amsterdam, The Netherlands). Data was synchronized and digitally stored at 400 Hz with Powerlab (AD Instruments, Oxford, UK) for offline analysis. After resting for 20 min, the vasoreactivity to carbon dioxide (VRCO_2_) and NVC protocols were performed, as described below. CBFV envelopes were continuously registered and analyzed offline.

### VRCO_2_

Participants were monitored through successive 2-min steps of resting, inhalation of a mixture of 5% CO_2_ and 95% O_2_ mixture (EtCO_2_ 7–10 mmHg above baseline), resting (room air, until normocapnia) and hyperventilation (EtCO_2_ 7–10 mmHg below baseline). VRCO_2_ was calculated as the slope of the relationship between EtCO_2_ average values plotted against those of relative CBFV achieved at the three stages, expressed as % mean CBFV per mmHg EtCO_2_ (Madureira et al., 2017).

### NVC

NVC was assessed in the PCA territory by a visual paradigm consisting of 10 cycles, each with a 20s resting phase (eyes closed) and 40 s stimulating phase (flickering checkerboard) at 10 Hz (Rosengarten et al., 2001a). The 5s of stable measurement prior to stimulation were used as the baseline (Rosengarten et al., 2001a). All cycles were synchronized and averaged. Peak systolic data was used because it is less prone to artifacts (Rosengarten et al., 2001b). Maximal systolic CBFV change was obtained to calculate the overshoot parameter as $\frac{maximumCBFV-baselineCBFV}{baselineCBFV}$ × 100%.[12] The systolic CBFV curve was modeled into a second order linear system to describe the dynamics of NVC response in time according to the equation *G*(*s*) = $\frac{K\;\times \;\left(1\;+\;Tvs\right)}{\frac{s2}{\omega 2}+2\xi *\frac{s}{\omega }+1}$, where “K” stands for gain, “Tv” for rate time, “ω” for natural frequency, and “ξ” for attenuation (Rosengarten et al., 2001a). All the parameters of the equation were determined by the least squares method. The sum of the squared residuals and the χ2 were also calculated to ensure the goodness of fit into the real measured values, as provided by the lsqnonlin function. Gain describes the relative CBFV difference between rest and steady-state level during visual stimulation; rate time indicates the initial steepness of the CBFV increase; natural frequency represents the oscillatory properties of the system; and attenuation describes dampening and tonus features, such as elastic properties of the vessel wall (Rosengarten et al., 2003). For this specific test, the MCA recordings were used as a control to detect non-specific changes in CBFV during the visual stimulation task.

### MRI Imaging

**Figure 1:**
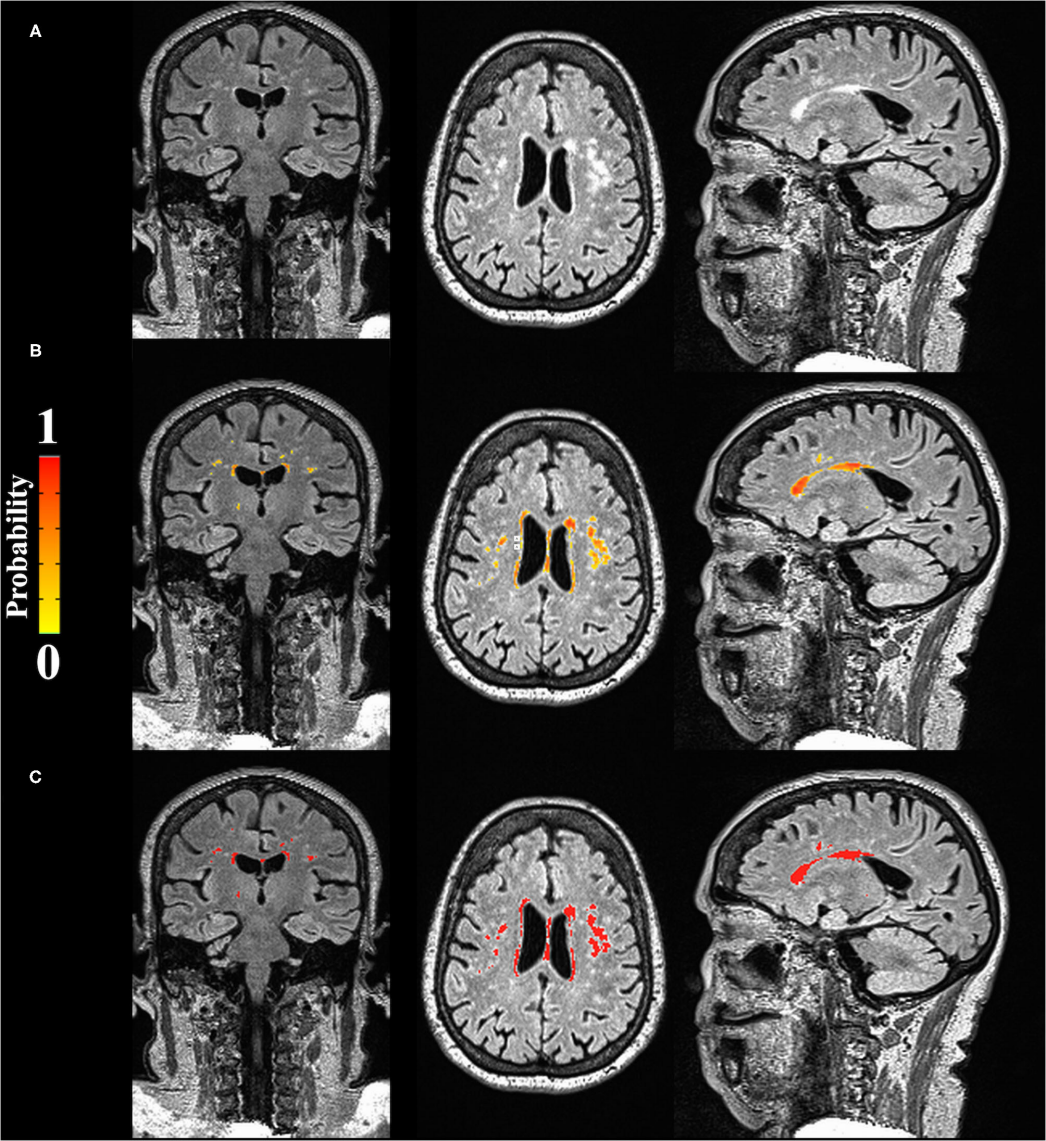
Automatic segmentation of WMH **(A)** Raw data; **(B)** Probability map of WMH; **(C)** Binarized results of WMH (values above 0 were considered WMH).

Forty-six patients were eligible and agreed to undergo cerebral MRI (Siemens Aera 1.5T). Of these, data from 6 patients were excluded for lack of quality for the evaluations. White matter hyperintensity (WMH) volumes normalized by intracranial volume were derived from the T2-weighted fluid-attenuated inversion recovery sequences collected in the sagittal plane. Voxels resolution was 1 x 1 x 1, slices = 256, FOV = 256 mm, TR = 5000 ms, TE = 336 ms, TI = 1800 ms (Figure 1). Briefly, WMH masks were created using the Lesion Segmentation Algorithm (LPA, 1) from the Lesion Segmentation Toolbox for SPM12 in MATLAB R2018a. Following an initial segmentation of the FLAIR image, probability maps were binarized using AFNI (2,3, v21.0.15) command 3dcalc. Resulting segmentations were quality-checked for sufficient accuracy and volumes were calculated using Freesurfer (v7.1) command mri_segstats.

Additional signs of CSVD were evaluated by an experienced vascular Neurologist to further characterize the CSVD in the patient cohort. Enlarged perivascular spaces (PVS) were defined as small (<0.3 mm) punctate or linear (if perpendicular or longitudinal to the plane of the scan, respectively) hyperintensities on T2 images in the basal ganglia (BG) and centrum semiovale (CS) (Potter et al., 2015). The PVS burden was then stratified into three groups: <11, 11–20 and >20 (Lau et al., 2017). Lacunes were defined as rounded or ovoid lesions >3 mm and <20 mm in diameter in the BG, internal capsule, CS or brainsteam, with CSF density on T2 images (Wardlaw et al., 2013). Cerebral microbleeds were defined as round, hypodense lesions <10 mm on susceptibility weighted imaging collected in the axial plane (slice thickness 2 mm, slices = 256, FOV = 230 mm, TR = 49 ms, TE = 40 ms), according to the guidelines (Greenberg et al., 2009).

### Statistical Analysis

Normality was determined using the Shapiro–Wilk test and analysis of skewness. Data with high asymmetry was normalized using logarithmic transformation. Homogeneity of variances was tested for each analysis. Baseline characteristics were compared using the independent sample *t*-test and χ2-test. Mixed ANOVA or ANCOVA (for PCA) were used to compare hemodynamic data. Subgroup analyses were performed using univariate ANOVA, with Bonferroni *post-hoc* tests. The partial eta squared (ηp^2^) was used as a measure of effect size: ηp^2^ > 0.14 indicates a large effect, ηp^2^ >0.06 indicates a medium effect and ηp^2^ > 0.01 indicates a small effect (Cohen, 1998). Age and gender were used as covariates in the ANOVA and ANCOVA comparisons. Supplemental analysis using body mass index (BMI) and VCS as covariates (plus age and gender) were also performed. Patients were dichotomized by the median value of WMH volume to compare the results of VRCO_2_ and NVC in the PCA between the two WMH burden groups. Subgroups were compared using the independent sample *t*-test.

Values of *p* < 0.05 were considered significant.

## Results

Fifty-two hypertensive patients were evaluated and TCD data from 17 healthy controls were used. Baseline characteristics are reported in Table 1. Supplementary File 1 depicts the burden of PVS, microbleeds and lacunes in the patient group. Most patients had low burden of PVS in the BG (55.0%) and CS (65.0%). Nine patients (22.5%) presented lacunes and four patients (10%) presented microbleeds.

**Table 1:** Demographics and baseline characteristics.

**Participant characteristics**	**Patients**	**Controls**	*p* value^*^
Age, years (mean ± SD)	64 ± 11	60 ± 16	0.376
Male, n (%)	28 (54)	8 (47)	0.627
BMI, kg/m^2^ (median ± IQR)	29 ± 5	25 ± 4	**<0.001**
Diabetes Mellitus, n (%)	30 (58)	0 (0)	
VCS, n (%)			
0	0 (0)	17 (100)	
1	2 (4)	0 (0)	
2	5 (10)	0 (0)	
3	22 (42)	0 (0)	
4	13 (25)	0 (0)	
5	8 (15)	0 (0)	
6	2 (4)	0 (0)	
HT duration, years (median ± IQR)	17 ± 6	0 (0)	
Chronic medication			
No. antihypertensives (median ± IQR)	3 ± 2	0 (0)	
ACEI/ARB, n (%)	48 (92)	0 (0)	
Diuretics, n (%)	39 (75)	0 (0)	
CCB, n (%)	37 (71)	0 (0)	
BB, n (%)	14 (27)	0 (0)	
Alpha2-agonists, n (%)	4 (8)	0 (0)	
Antiplatelets, n (%)	14 (27)	0 (0)	
Statins, n (%)	40 (77)	0 (0)	
Cognitive parameters (median ± IQR)			
Education, years	4 ± 5		
MMSE	28 ± 3		
MoCA	22 ± 5		
ABPM, mmHg (median ± IQR)			
24-h systolic BP	130 ± 15	-	-
24-h diastolic BP	78 ± 13	-	-
24-h pulse pressure	57 ± 16	-	-
Finapres BP, 5 min (mean ± SD)			
Systolic BP	136 ± 24	109 ± 25	**<0.001**
Diastolic BP	62 ± 17	50 ± 14	**0.035**
Mean BP SP, mmHg_2_ (median ± IQR)	9 ± 24	7 ± 8	**0.007**
EtCO_2_, mmHg (median ± IQR)	36 ± 4	38 ± 3	0.553

* *Values were obtained using the t-test or the χ2-test. ABPM, ambulatory blood pressure monitoring; BMI, body mass index; BP: blood pressure; EtCO_2_, end-tidal carbon dioxide; IQR, interquartile range; SD, standard deviation; SP, spectral power; VCS, vascular comorbidity score. Bold values of statistically significant p values (p < 0.05)*.

### Cerebral Hemodynamics and VRCO_2_

Baseline CBFV and CBFV variability (spectral power) were similar in patients and controls (Table 2). VRCO_2_ in either territory (PCA or MCA) was similar between groups and showed no change after adjusting for both VCS and BMI (Supplementary File 2).

**Table 2:** Cerebral hemodynamics, VRCO_2_ and NVC: patients vs. controls, controlling for age and gender.

	**Patients**		**Controls**		**Artery**	**Group**	**Interaction**
	**MCA**	**PCA**	**MCA**	**PCA**	*p* value^*^	*p* value^*^	*p* value^*^
**Cerebral hemodynamics**							
Mean CBFV (cm/s)	47.6 ± 13.4^†^	31.4 ± 8.2^†^	52.4 ± 16.5^†^	29.1 ± 10.1^†^	0.044	0.925	0.161
MFV SP (cm/s^2^)	3.3 ± 5.0^‡^	2.5 ± 4.7^‡^	3.8 ± 4.3^‡^	2.3 ± 3.1^‡^	0.976	0.770	0.119
**VRCO**_**2**_ (%/mmHg CO_2_)	1.4 ± 0.5^†^	0.9 ± 0.4^‡^	1.7 ± 0.6^†^	1.0 ± 0.7^‡^	0.107	0.588^#^	0.437
**Neurovascular coupling**							
Overshoot^§^ systolic CBFV (%)		22.4 ± 9.2^†^		31.6 ± 5.7^†^		**<0.001**	
Modeled parameters							
Gain (%)		14.0 ± 7.1^†^		17.3 ± 4.8^†^		0.118	
Natural frequency (Hz)		0.18 ± 0.08^‡^		0.22 ± 0.06^‡^		**<0.001**	
Attenuation (a.u)		0.4 ± 0.4^‡^		0.4 ± 0.4^‡^		0.374	
Rate time (s)		0.00 ± 0.02^‡^		0.03 ± 6.81^‡^		**0.001** ^**#**^	

* *Two-factor mixed-design ANOVA for the interaction between group variable (patients vs. controls) and arterial territory (MCA vs. PCA), controlling for age and gender. For NVC, values were obtained using an ANCOVA. Effect size: rate time ηp^2^ = 0.174, natural frequency ηp^2^ = 0.237 and overshoot systolic CBFV ηp^2^ = 0.186. ^||^gender significantly interfered with the model;*

# *age significantly interfered with the model;*

† *Values are presented as mean ± standard deviation*.

‡ *Values are presented as median ± interquartile range*.

§ *Maximal CBFV increase during visual stimulation. a.u., arbitrary units; CBFV, cerebral blood flow velocity; VRCO_2_, vasoreactivity to carbon dioxide; MFV SP, median flow velocity spectral power; MCA, middle cerebral artery; PCA, posterior cerebral artery. Bold values of statistically significant p values (p < 0.05)*.

There were no differences in VRCO_2_ between hypertensive non-diabetics (HT-nDM), hypertensive diabetics (HT-DM) and controls (Table 3). Those results did not change significantly after controlling for BMI or VCS (Supplementary File 3).

**Table 3:** Cerebral hemodynamics, VRCO_2_ and NVC: controls vs. HT-nDM vs. HT-DM patients, controlled for age and gender.

	**HT-nDM**		**HT-DM**		**Controls**		**Group**	**HT-nDM vs. Controls**	**HT-DM vs. Controls**	**HT-nDM vs. HT-DM**
	**MCA**	**PCA**	**MCA**	**PCA**	**MCA**	**PCA**	*p* value^*^	*p* value^*^	*p* value^*^	*p* value^*^
**Cerebral hemodynamics**										
Mean CBFV (cm/s)^†^	47.0 ± 14.1	33.4 ± 8.4	48.0 ± 13.2	30.0 ± 7.8	52.4 ± 16.5	29.1 ± 10.1	0.993			
MFV SP (cm/s^2^)^‡^	3.1 ± 4.7	2.6 ± 5.0	3.7 ± 7.0	2.5 ± 5.0	3.8 ± 4.3	2.3 ± 3.1	0.921			
**VRCO**_**2**_ (%/mmHg CO_2_)^†^	1.4 ± 0.6	1.2 ± 0.7	1.4 ± 0.5	0.8 ± 0.3	1.7 ± 0.6	1.1 ± 0.4	0.676^#^			
**Neurovascular coupling**										
Overshoot^§^ systolic CBFV (%)^†^		25.1 ± 8.6		20.7 ± 9.3		31.6 ± 5.7	**<0.001**	0.080	**<0.001**	0.248
Modeled parameters										
Gain (%)^†^		13.9 ± 5.1		14.1 ± 8.2		17.3 ± 4.8	0.283			
Natural frequency (Hz) ^†^		0.19 ± 0.04		0.16 ± 0.05		0.23 ± 0.06	**<0.001** ^#^	0.052	**<0.001**	**0.043**
Attenuation (a.u)^†^		0.4 ± 0.3		0.4 ± 0.3		0.5 ± 0.2	0.327^#^			
Rate time (s)^‡^		0.00 ± 0.00		0.00 ± 0.26		0.03 ± 6.81	**0.005** ^#^	**0.012**	**0.011**	1.000

* *Two-factor mixed-design ANOVA for the interaction between group variable (HT-DM vs. HT-nDM vs. controls) and arterial territory (MCA vs. PCA), controlling for age and gender, with Bonferroni post-hoc. For NVC, values were obtained using an ANCOVA. Effect size: rate time ηp^2^ = 0.174, natural frequency ηp^2^ = 0.313 and overshoot systolic CBFV ηp^2^ = 0.22*.

† *Values are presented as mean ± standard deviation*.

‡ *Values are presented as median ± interquartile range*.

§ *Maximal CBFV increase during visual stimulation. ^||^gender significantly interfered with the model*.

# *age significantly interfered with the model. a.u., arbitrary units; CBFV, cerebral blood flow velocity; HT-DM, hypertensive diabetic; HT-nDM, hypertensive non-diabetic; MCA, middle cerebral artery; MFV SP, median flow velocity spectral power; PCA, posterior cerebral artery; VRCO_2_, vasoreactivity to carbon dioxide. Bold values of statistically significant p values (p < 0.05)*.

### NVC

**Figure 2:**
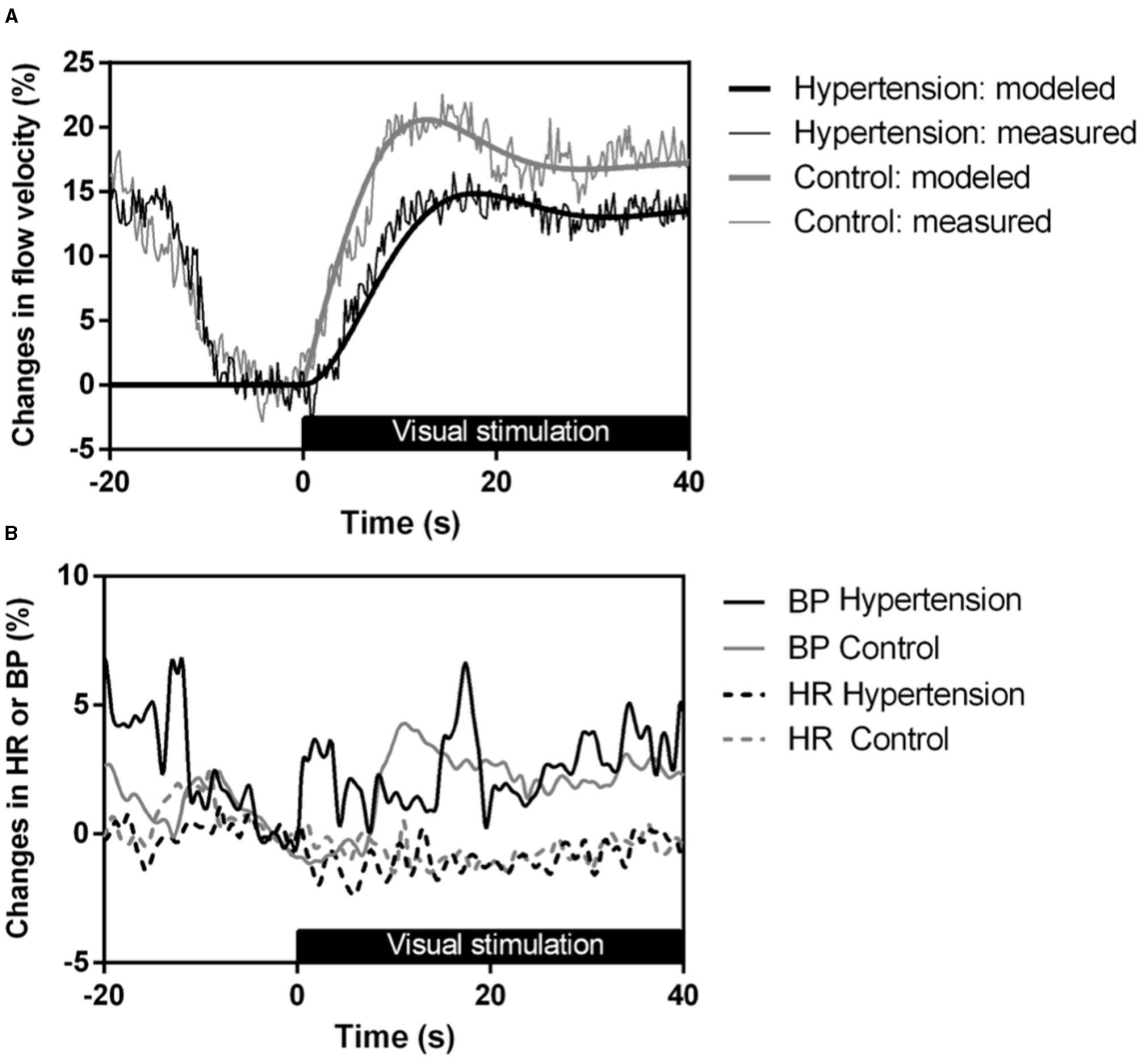
**(A)** Group-averaged evoked systolic CBFV responses during visual stimulation with the flickering checkerboard; **(B)** Blood pressure (BP) and heart rate (HR) during visual stimulation with the flickering checkerboard. Gray lines represent healthy controls and black lines represent HT patients (thin lines: measured responses, thick lines: modeled blood flow data).

NVC in the PCA territory was significantly altered in HT patients, with smaller increases in CBFV during visual stimulation (*p* < 0.001) and disturbed NVC time-varying properties, with lower natural frequency (*p* < 0.001) and lower rate time (*p* = 0.001) (Table 2; Figure 2). These results remained similar when adjusting for BMI and VCS, although the overshoot systolic CBFV was over the limit of statistical significance when adjusting for the VCS (*p* = 0.065) (Supplementary File 2).

NVC results were worse in HT-DM than HT-nDM (Table 3). For the overshoot systolic CBFV and for natural frequency, only patients with both comorbidities showed significant differences when comparing to controls. HT-DM showed lower natural frequency than non-diabetic patients (*p* = 0.043). When controlling for VCS, natural frequency was also worse in both HT-nDM (*p* = 0.020) and HT-DM (*p* = 0.002) when comparing to controls, with the worst results for HT-DM (HT-nDM vs. HT-DM, *p*=0.011) (Supplementary File 3).

**Figure 3:**
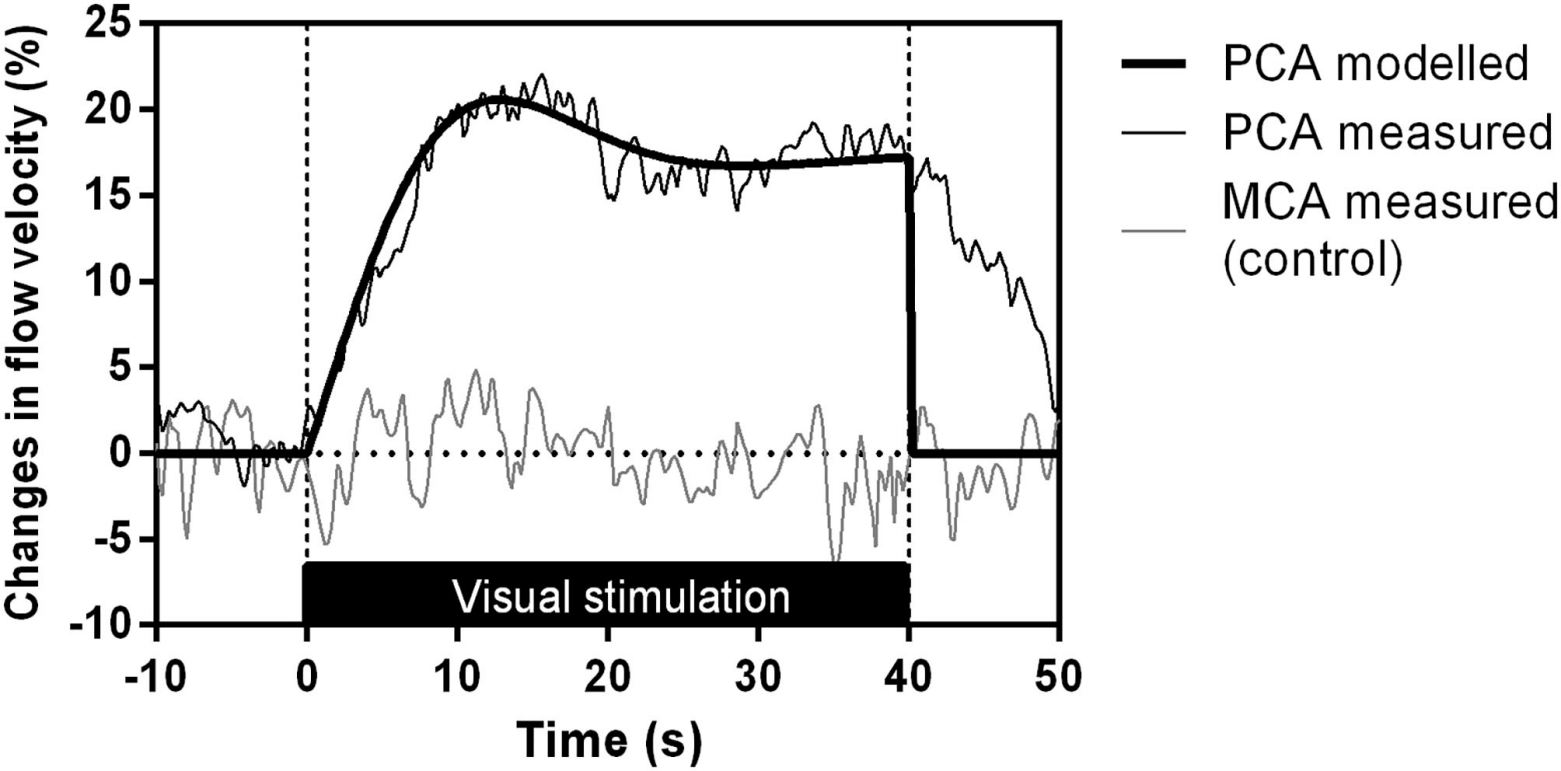
Evoked systolic CBFV responses in the PCA and MCA during visual stimulation with the flickering checkerboard in one HT patient. The black lines represent the PCA response (thin lines: measured responses, thick lines: modeled blood flow data) and the gray lines represent the MCA measured response, used as a control.

Figure 3 represents the evoked systolic CBFV responses in the PCA and MCA during the visual stimulation in one HT subject, to demonstrate the individual NVC responses.

### Association With White Matter Hyperintensities

As shown in Table 4, VRCO_2_ was lower in the higher burden group in both the MCA (*p* = 0.004) and the PCA (*p* = 0.007). NVC parameters did not differ in both groups.

**Table 4:** NVC and VRCO_2_ in relation to the WMH volume in hypertensive patients.

	WMH volume^*^					
	**MCA**			**PCA**		
	**≤ 0.14**	**> 0.14**	***p* value**	**≤ 0.14**	**> 0.14**	***p* value**
**VRCO**_**2**_ (%/mmHg CO_2_)	1.6 ± 0.5	1.2 ± 0.5	**0.004**	1.0 ± 0.6^‡^	0.8 ± 0.5^‡^	**0.006**
**Neurovascular coupling**						
Overshoot^§^ systolic CBFV, (%)	-	-	-	23.4 ± 10.0^†^	21.8 ± 8.8^†^	0.573
Gain (%)	-	-	-	11.7 ± 11.1^‡^	14.6 ± 7.9^‡^	0.297
Natural frequency (Hz)	-	-	-	0.2 ± 0.0^†^	0.2 ± 0.1^†^	0.432
Attenuation (a.u)	-	-	-	0.4 ± 0.3^†^	0.4 ± 0.2^†^	0.891
Rate time (s)	-	-	-	0.0 ± 1.2^‡^	0.0 ± 0.0^‡^	0.169

* *Dichotomized by median values. p values were obtained using the t-test*.

† *Values are presented as mean ± standard deviation;*

‡ *Values are presented as median ± interquartile range*.

§ *Maximal CBFV increase during visual stimulation. a.u., arbitrary units; CBFV, cerebral blood flow velocity; MCA, median cerebral artery; PCA, posterior cerebral artery; VRCO_2_, vasoreactivity to carbon dioxide; WMH, white matter hyperintensities. Bold values of statistically significant p values (p < 0.05)*.

## Discussion

Our study shows that NVC was significantly impaired in hypertensive patients, with reduced CBFV increase and altered time behavior hemodynamic evoked response during visual stimulation. Moreover, NVC tended to be worse in the DM subgroup. VRCO_2_ remained relatively preserved. These results did not change when adjusting for other vascular risk factors. The novel finding is that the natural frequency seems to be the most sensitive parameter for discriminating abnormal NVC in these patients. Rosengarten and colleagues reported that this parameter had the lowest SD of the modeled parameters, thus having the potential for better differentiating between normal and abnormal NVC, but its capacity to identify vascular response dysfunction in disease settings needed to be studied (Rosengarten et al., 2001a). Our results seem to point toward natural frequency as a possible marker for NVC dysfunction in hypertension and diabetes.

Overall, the white matter disease (WMD) burden was low in the patient cohort. The NVC results were similar between higher and lower WMD burden groups, while higher burden patients showed worse VRCO_2_. It has been recently reported that reduced VRCO_2_ precedes the development of WMH (Sam et al., 2016), which could help explain the difference between the disease burden groups. Similar NVC with higher and lower WMH volume has been previously reported (Sorond et al., 2013). Although WMH volume predicts an increased risk of stroke and cognitive decline, the clinical expression seems to vary (Sorond et al., 2011). Moreover, silent markers of CSVD are frequently detected on the MRI of older individuals without cognitive impairment (Vernooij et al., 2009; Debette and Markus, 2010). Thus, besides macrostructural changes, other modalities reflecting microstructural integrity and function, such as TCD dynamic studies, may provide additional information to further stratify patients at risk.

### Neurovascular Unit at the Core of Target Brain Damage in Hypertension

Our results showed a significantly reduced PCA CBFV magnitude response and altered time behavior of reactive hyperemia during visual stimulation in HT patients, especially when diabetes was an added comorbidity. These findings indicate disturbed NVC in these patients' PCA cortical territory, independently of established WMD, as was observed by other groups (Birns et al., 2009; Purkayastha et al., 2014). The decreased CBFV during visual stimulation reflects less robust functional hyperemia, causing failure to meet the metabolic demands and neuronal damage (Iadecola et al., 2019).

The reduced overshoot systolic CBFV in the patient group was not accompanied by a reduction in the modeled parameter gain. This could reflect lack of statistical power to detect the differences between the groups, since the mean gain was lower in the patient group. In addition, it has been demonstrated that the overshoot of systolic CBFV is significantly influenced by not only gain, but also rate time and attenuation (Rosengarten et al., 2001a). Hence, the parameter gain and the overshoot systolic CBFV may not be perfectly matched.

These results are in line with studies on genetically determined CSVD. CADASIL patients demonstrated less robust functional hyperemia in the PCA during visual stimulation, with changes in time dynamics, very similar to our findings (Jokumsen-Cabral et al., 2019). Comparable NVC dysfunction in the posterior circulation was shown in Fabry patients (Azevedo et al., 2012; Castro et al., 2020). Both diseases are characterized by abnormal material deposition in the vessel walls (Baudrimont et al., 1993; Rombach et al., 2010). Interestingly, amyloid deposition also seems to cause NVC impairment (Iadecola and Davisson, 2008; Brickman et al., 2015), and HT appears to have a role in promoting amyloid deposition, thus working synergistically to worsen CSVD and cognitive decline (Iadecola and Davisson, 2008).

Chronic HT leads to structural (mal)adaptations in the cerebral circulation, with remodeling of the cerebral arteries and arterioles. This remodeling involves smooth muscle cell hypertrophy and hyperplasia, increased deposition of extracellular matrix components and degenerated smooth muscle (lipohyalinosis) and fibrinoid necrosis, leading to arterial stiffening and loss of elasticity (Iadecola and Gottesman, 2019). These changes in the proximal resistance arteries cause substantial burden on the vulnerable downstream microcirculation, promoting pressure-induced oxidative stress to the endothelial cells and neuroinflammation (Ungvari et al., 2021). In experimental models, HT results in impairment of endothelium mediated neurovascular coupling responses, in part resulting from this oxidative stress and neuroinflammation (Iadecola and Gottesman, 2019; Ungvari et al., 2021). Hence, the structural changes induced by HT play an important role in the loss of functional integrity of the neurovascular unit. Natural frequency is assumed to represent the tonus and the speed of the system (Rosengarten et al., 2003), which would be altered by the increased rigidity of the vessels and endothelial dysfunction, and our results show that this parameter was the most sensitive in differentiating patients from controls.

Less effective NVC in the MCA territory has been associated with significant cognition, balance, and walking velocity changes in the elderly (Sorond et al., 2011; Purkayastha et al., 2014). Despite the presence and burden of WMD, normal NVC was associated with preserved walking speed, while slower walking is one of the earliest manifestations of CSVD. Further prospective work could use NVC for predicting symptomatic CSVD. Curiously, cocoa and deferoxamine have been demonstrated to reverse some of these changes suggesting the possibility for pharmacological modulation of the neurovascular function (Sorond et al., 2013, 2015).

### Additional Contribute From Diabetes Mellitus

Comorbid diabetes associated with increased cerebrovascular dysfunction. NVC was worse in this subgroup of hypertensive patients, particularly in its oscillatory properties (natural frequency), in accordance to previous studies in early type 1 DM (Rosengarten et al., 2002). This might signify higher rigidity of the small arteries due to the accumulation of advanced glycated by-products. In fact, type 2 DM patients have particularly high incidence of lacunar stroke (van Harten et al., 2006; Brundel et al., 2014). Diabetes induced chronic vascular changes include not only macrovascular disorders, such as cardiovascular and cerebrovascular large vessel disease, but also microvascular disorders, with nephropathy, retinopathy and neuropathy (Chawla et al., 2016). Furthermore, studies have implicated DM as a risk factor for cognitive impairment, which may be related to CSVD. However, the mechanism by which cognitive decline occurs and whether it can be explained by dysfunction of the neurovascular unit remains to be elucidated (Mogi and Horiuchi, 2011). The sympathetic nervous system seems to attenuate the cerebrovascular response to hypercapnia, suggesting a direct effect on the cerebral vasculature (Jordan et al., 2000). NVC is also affected by autonomic dysfunction (Azevedo et al., 2011). Thus, differences in DM vs. nDM hypertensive patients could be related to DM associated dysautonomia in CSVD.

Overall, our study further supports cerebrovascular dynamics dysfunction as a major player in explaining the relationship between increased VRF burden and CSVD manifestations, independently of macroscopic white matter lesions.

### Limitations

We acknowledge several methodological limitations. Due to the cross-section design, our study cannot provide evidence to support cerebrovascular dysfunction as an early predictor of CSVD. However, these patients had well-controlled HT, based on average ABPM values, with no clinical manifestations of cerebrovascular disease. All the patients were referred to the Hypertension Unit due to severe or difficult to manage HT, and we do not know the duration of the untreated disease, which could impact the degree of microvascular dysfunction.

Although the patient group's sample size is relatively large for hemodynamic physiology studies, this is an exploratory study and must be validated in larger, multicenter cohorts. Also, the control group is relatively small, not exactly age- and gender-matched, and the differences in comorbidities between the two groups can be potential confounders to the observed differences. Besides HT and DM, already discussed, other vascular comorbidities have been associated with the development of CSVD. Dyslipidemia plays an important role in the development of large vessel disease and stroke, but its role in CSVD is still controversial (Tsai et al., 2018). However, animal studies have demonstrated cerebral autoregulation impairment with hyperlipidemia and a relationship between hyperlipidemia and the development of CSVD (Ayata et al., 2013; Kraft et al., 2017). Obesity has been demonstrated to have an impact in the development of CSVD (Yamashiro et al., 2014), and it has been shown to affect cerebral vasoreactivity (Selim et al., 2008). Smoking appears to worsen the effects of hypertension in the cerebral microvasculature (Hara et al., 2019), and there is impaired neurovascular coupling in the PCA of young chronic smokers (Olah et al., 2008). Chronic heart failure can affect cerebral autoregulation, reduce cerebral blood flow (CBF) and has been shown to affect NVC in the PCA (Aires et al., 2020; Ovsenik et al., 2021). The prevalence of CSVD is higher in patients with coronary artery disease (Berry et al., 2019), and it has been identified as an independent risk factor for vascular dementia (Gorelick et al., 2011). It was recently demonstrated that aTrial fibrillation reduces cerebral autoregulation and impairs neurovascular coupling responses to visual stimulation (Junejo et al., 2020). There is evidence that peripheral artery disease is associated with white matter disease and the development of vascular dementia (Bots et al., 1993; Gorelick et al., 2011). In patients with ischemic stroke, impaired renal function correlated with worse dCA and associated with an increased risk of hemorrhagic transformation (Castro et al., 2018). However, in addition to adjusting for age and gender, adjusting for the vascular comorbidities did not modify the results significantly.

Hypertension affects cerebral small vessels heterogeneously, and MRI seems the logical choice for its superior spatial resolution. However, MRI VRCO_2_ protocols are more prone to failure (Moreton et al., 2018), expensive and not as standardized as TCD (Malojcic et al., 2017). Moreover, TCD offers extraordinary time resolution (~5 ms) for studying the time behavior of CBFV activation in downstream cortical microvasculature. There are also inherent limitations for continuous blood flow monitoring by TCD, namely, providing a measure of blood flow velocity and not flow. However, the former is an adequate surrogate for the latter as long as the insonated artery diameter remains constant. Since data were acquired in a supine position and relied on spontaneous measurements, changes in artery diameter are not anticipated for NVC. Nevertheless, it has been demonstrated that with increasing partial pressure of arterial CO_2_ there is noticeable increase in vessel diameter, which could lead to underestimation of cerebral blood flow in the VRCO_2_ protocol (Coverdale et al., 2014).

We did not report the presence of cortical microinfarcts (CMI) in this study, despite having been found in cohorts of hypertensive patients (although not consistently) and despite their importance in cognitive decline (van Veluw et al., 2017). We did not detect CMI upon visual inspection of the MRI scans. However, the protocols for detecting CMI are mostly validated in 3T MRI scanners (Coverdale et al., 2014), and our 1.5T scan protocol is underpowered for their detection.

In conclusion, neurovascular coupling, and more specifically the modeled parameter natural frequency, seems to be particularly affected in hypertension and diabetes and could be useful as an early biomarker for microvascular dysfunction, future irreversible vascular damage, and cognitive decline. Additionally, our study supports TCD dynamic tests as useful tools for better understating microvascular damage associated with these diseases, but future research is warranted to confirm these results.

## Data Availability Statement

The raw data supporting the conclusions of this article may be made available by the authors upon request, without undue reservation.

## Ethics Statement

The studies involving human participants were reviewed and approved by Comissão de Ética da U. L. S. Matosinhos, Hospital de Pedro Hispano, EPE, Unidade Local de Saúde de Matosinhos, Portugal. The patients/participants provided their written informed consent to participate in this study.

## Author Contributions

AM: conception and design, data collection, literature search, drafting the manuscript, and critical revision of the manuscript. PC: conception and design, data collection, critical revision of the manuscript, and supervision. GP, CF, and JP: data collection and critical revision of the manuscript. FS, AM, and JH: critical revision of the manuscript. EA: conception and design, critical revision of the manuscript, and supervision. All authors contributed to the article and approved the submitted version.

## Funding

This study received financial support from Associação para o Estudo das Doenças Neurovasculares for performing MRI studies.

## Conflict of Interest

The authors declare that the research was conducted in the absence of any commercial or financial relationships that could be construed as a potential conflict of interest.

## Publisher's Note

All claims expressed in this article are solely those of the authors and do not necessarily represent those of their affiliated organizations, or those of the publisher, the editors and the reviewers. Any product that may be evaluated in this article, or claim that may be made by its manufacturer, is not guaranteed or endorsed by the publisher.
