# Supplementary material for: Neurovascular Coupling Is Impaired in Hypertensive and Diabetic Subjects Without Symptomatic Cerebrovascular Disease
Source: Front Aging Neurosci. 2021 Oct 6;13:728007. doi: 10.3389/fnagi.2021.728007 (PMC8526560; doi:10.3389/fnagi.2021.728007)
Supplement: Supplementary file 1 [file Data_Sheet_1.docx]

Supplementary Material

# Supplementary Figures and Tables

Supplementary file 1. Imaging characteristics of the patient group

| **Imaging characteristics** | **N (%)** |
| --- | --- |
| Basal ganglia PVS |  |
| <10 | 22 (55) |
| 10-20 | 8 (20) |
| >20 | 10 (25) |
| Centrum semiovale PVS |  |
| <10 | 26 (65) |
| 10-20 | 7 (17.5) |
| >20 | 7 (17.5) |
| Lacunes |  |
| Present | 9 (22.5) |
| Absent | 31 (77.5) |
| Microbleeds |  |
| Present | 4 (10.0) |
| Absent | 36 (90.0) |
| Strictly deep | 2 (5.0) |
| Strictly lobar | 1 (2.5) |
| Mixed location | 1 (2.5 |

| PVS: perivascular spaces |
| --- |

Supplementary file 2. Cerebral hemodynamics, VRCO_2_ and NVC: patients versus controls (controlling for age, gender and BMI or age, gender and VCS)

|  | **Age + Gender + BMI** | | | **Age + Gender + VCS** | | |
| --- | --- | --- | --- | --- | --- | --- |
|  | **Artery** | **Group** | **Int.** | **Artery** | **Group** | **Int.** |
|  | *p* value^*^ | *p* value^*^ | *p* value^*^ | *p* value^*^ | *p* value^*^ | *p* value^*^ |
| **Cerebral hemodynamics** | | | | | | |
| Mean CBFV (cm/s) | 0.210 | 0.718 | 0.171 | 0.070 | 0.396 | 0.146 |
| MFV SP (cm/s^2^) | 0.745 | 0.333 | 0.472 | 0.878 | 0.935 | 0.107 |
| **VRCO_2_** (%/mmHg CO_2_) | **0.008** | 0.840^\|\|^ | 0.908 | 0.144 | 0.581^\|\|^ | 0.141 |
| **Neurovascular coupling** | | | | | | |
| Overshoot^†^ systolic CBFV (%) |  | **0.002** |  |  | 0.065 |  |
| *Modeled parameters* | | | | | | |
| Gain (%) |  | 0.117 |  |  | 0.926 |  |
| Natural frequency (Hz) |  | **0.001** |  |  | **0.046** |  |
| Attenuation (a.u) |  | 0.312 |  |  | 0.210^\|\|^ |  |
| Rate time (s) |  | **0.012**^\|\|^ |  |  | **0.047**^\|\|^ |  |
| *Two-factor mixed-design ANOVA for the interaction between group variable (patients vs controls) and arterial territory (MCA vs PCA), controlling for age and gender, age, gender and BMI or age, gender and VCS. For NVC, values were obtained using an ANCOVA. Effect size: BMI - rate time ηp² = 0.109, natural frequency ηp²=0.176 and overshoot systolic CBFV ηp²= 0.140; VCS - rate time ηp² = 0.068 and natural frequency ηp²=0.069. †Maximal CBFV increase during visual stimulation. §gender significantly interfered with the model. \|\|age significantly interfered with the model. a.u.: arbitrary units; BMI: body mass index; CBFV: cerebral blood flow velocity; Int: interaction; MCA: middle cerebral artery; MFV SP: median flow velocity spectral power; PCA: posterior cerebral artery; VCS: vascular comorbidities score; VRCO_2_: vasoreactivity to carbon dioxide. | | | | | | |

Supplementary file 3. Cerebral hemodynamics, VRCO2 and NVC: controls versus HT-nDM versus HT-DM patients (controlling for age and gender, age, gender and BMI or age, gender and VCS)

|  | **Age + Gender +BMI** | | | | **Age + Gender + VCS** | | | | |
| --- | --- | --- | --- | --- | --- | --- | --- | --- | --- |
|  | **Group** | **HT-nDM vs controls** | **HT-DM vs controls** | **HT-nDM vs HT-DM** | **Group** | **HT-nDM vs controls** | **HT-DM vs controls** | **HT-nDM vs HT-DM** |  |
|  | *p* value^*^ | *p* value^*^ | *p* value^*^ | *p* value^*^ | *p* value^*^ | *p* value^*^ | *p* value^*^ |  |  |
| **Cerebral hemodynamics** | | | | | | | | |  |
| Mean CBFV (cm/s) | 0.936 |  |  |  | 0.565 |  |  |  |  |
| MFV SP (cm/s^2^) | 0.621 |  |  |  | 0.987 |  |  |  |  |
| **VRCO_2_ (%/mmHg CO2)** | 0.910^\|\|^ |  |  |  | 0.860^\|\|^ |  |  |  |  |
| **Neurovascular coupling** | | | | | | | | |  |
| Overshoot^†^ systolic CBFV (%) | **0.002** | 0.126 | **0.002** | **0.249** | **0.022** | 0.071 | **0.018** | 0.118 |  |
| *Modeled parameters* | | | | | | | | |  |
| Gain (%) | 0.287 |  |  |  | 0.558 |  |  |  |  |
| Natural frequency (Hz) | **<0.001**^\|\|^ | 0.090 | **<0.001** | 0.067 | **0.002**^\|\|^ | **0.020** | **0.002** | **0.011** |  |
| Attenuation (a.u) | 0.323^\|\|^ |  |  |  | 0.333^\|\|^ |  |  |  |  |
| Rate time (s) | **0.043**^\|\|^ | 0.054 | 0.104 | 1.000 | 0.142^\|\|^ |  |  |  |  |
| *Two-factor mixed-design ANOVA for the interaction between group variable (HT-nDM vs HT-DM vs controls) and arterial territory (MCA vs PCA), controlling for age, gender and BMI or age, gender and VCS, with Bonferroni *post-hoc*. For NVC, values were obtained using an ANCOVA, with Bonferroni *post-hoc*. Effect size: BMI - natural frequency ηp²=0.249 and overshoot systolic CBFV ηp²= 0.180; VCS - natural frequency ηp²=0.198 and overshoot systolic CBFV ηp²= 0.119. †Maximal CBFV increase during visual stimulation. §gender significantly interfered with the model. \|\|age significantly interfered with the model. a.u.: arbitrary units; BMI: body mass index; CBFV: cerebral blood flow velocity; MCA: middle cerebral artery; MFV SP: median flow velocity spectral power; PCA: posterior cerebral artery; VCS: vascular comorbidities score; VRCO_2_: vasoreactivity to carbon dioxide. | | | | | | | | |  |
